# Formaldehyde as a Disinfectant

**Published:** 1898-02

**Authors:** 


### FORMALDEHYDE AS A DISINFECTANT.

So much has appeared in recent medical literature concerning the use of this agent, and so many claims have been made in regard to it, some of which evidently have not been founded upon painstaking investigation, that it is well for us to review the matter carefully to find out just what position formaldehyde should occupy as an agent for disinfection. As with all new remedies of value extravagant claims are made in regard to it. There seems to be no doubt as to its value as a surface disinfectant. There has been considerable variation in opinion as to its power of penetration. The opinion in regard to these points is somewhat easily biased by the claims of those who have certain forms of apparatus for generating and distributing gas.

Dr. Charles Harrington, Instructor in Hygiene and Materia Medica in the Harvard Medical School, reports in the *American Journal of Medical Sciences* a series of experiments to demonstrate the bactericidal and penetrative powers of this agent. The experiments were conducted in two of the surgical operating rooms and in the pathological laboratory of the Boston City Hospital. The cultures employed were stapoylococcus aureus, typhoid, anthrax spores, a non-pathological spore bearer, dust from shelf and diphtheria throat culture. While we can not detail the several experiments, attention can be called to the conclusions, which seem reasonably based upon these. As a surface disinfectant, Dr. Harrington claims that formaldehyde has greater power than any other substance. It is, however, not absolutely thorough in all cases as a surface disinfectant, as was demonstrated in experiments for room disinfection. Ordinary bacteria, when freely exposed to an atmosphere produced by vaporizing approximately 110 c. c. of formalin in one thousand cubic feet of space, are killed within two and one-half hours. The penetrating power of the gas is largely dependent upon the conditions as to moisture. Through dry, pervious wrappings or substances, it penetrates with comparative ease, yet not always with sufficient germicida

If an increase in the amount of uric acid is observed, it should be ascertained whether this increase is only relative or absolute. It will readily be understood that the diagnosis of an absolute increase is only justifiable if the amount of urine is not subnormal. The increase, so frequently observed in febrile urines, is usually of a relative character, the total amount of urine being subnormal. A sharp line of distinction should, furthermore, be drawn between increased production and increased elimination. It is thus not justifiable to exclude the diagnosis "uric acid diathesis," when normal or even subnormal amounts are obtained. A temporary retention is frequently observed.

The subject of uric acid elimination is one of the most interesting and practically important in clinical medicine, and the writer would suggest this topic to his colleagues as a most promising one for careful investigation. It is a wide field, and one upon which the general practitioner, with very modest laboratory facilities even, may be able to accomplish a great deal of practical as well as theoretical value. It is thus quite likely that the constant elimination of relatively, not necessarily absolutely, increased amounts of uric acid will ultimately lead to definite anatomical alterations of the kidney structure, but the question still requires a great deal of careful investigation. The general practitioner can do more in this respect than the hospital physician, because he is able to follow his patient's history for a very much longer period of time.

An excess of urea is likewise quite readily discovered with the nitric acid test. Every physician probably, who has occasion to examine many urines, has observed the appearance of glistening crystals after the addition of the nitric acid, if the specimen has been allowed to stand for a few minutes. These crystals are urea nitrate, and when formed in this manner always indicate the presence of at least 25 grammes of urea for every 1,000 c. c. of urine. When occurring in dense masses 50 grammes or more are being eliminated.

In conclusion, the nitric acid test, when applied as described, indicates the presence or absence of bile pigments, as well as the presence of increased amounts of indican. A dark blue or violet ring is only found when indican is eliminated in large amounts, and as this, generally speaking, only occurs when an increased degree of intestinal putrefaction exists, we have thus a fairly accurate index by which to measure the latter.

It is thus seen that with this simple test a large amount of valuable information may be obtained, but the writer wishes to emphasize that the question of technique in clinical diagnosis is only of secondary importance. Above all the physician must learn to *interpret* his results.—*National Medical Review.*

